# Pod save you: assisting the transition to audio-based asynchronous learning

**DOI:** 10.5195/jmla.2021.1349

**Published:** 2021-10-01

**Authors:** Brandon Patterson, Bryan Elias Hull

**Affiliations:** 1 b.patterson@utah.edu, Technology Engagement Librarian, Eccles Health Sciences Library, University of Utah, Salt Lake City, Utah; 2 bryan.hull@utah.edu, Digital Publishing Program Manager, Eccles Health Sciences Library, University of Utah, Salt Lake City, Utah

## Abstract

**Background:**

In 2017, an academic health sciences library in Utah developed a multimedia studio for students, faculty, and academic staff. Educational projects needing video, audio, and lecture capture could utilize a one-button studio for recording video sessions, microphones for audio, and various screen capture software for lectures. Since the pandemic, this service has seen rapid growth due to academic lectures going exclusively online. In response, the library launched a dedicated podcasting suite to accommodate the increase in students and faculty needing to record lectures or podcasts for others in the medical profession.

**Description:**

This article will outline the process of creating the podcasting suite and provide equipment rosters and methods other libraries may consider for establishing their own studio. Administrating duties of the studio will also be included, such as handling reservations and user assessment. An instructional guide for users is also included to assist patrons in accomplishing their podcast creations.

**Conclusion:**

Podcasts created in the space range from topics about teaching strategies in medicine to diagnoses and treatments of skin disorders. A podcasting suite is another way libraries can provide valuable services for asynchronous learning and student projects. Students, staff, and faculty have appreciated the ease of the service and the support behind it. A feedback loop was developed to further improve the space to meet the needs of users.

In 2017, staff from the Eccles Health Sciences Library at the University of Utah converted a computer lab into a complete audio and visual recording studio where students, staff, and faculty were invited to create educational multimedia projects. The space, which came to be known as the Tree of Hippocrates Education Studio (THE Studio), was primarily used to record video sessions, with limited capability to record audio-based projects and lecture capture. As the COVID-19 pandemic struck, staff saw an increase in demand for these recording resources as academic lectures transitioned to online. Audio-based projects were especially in demand and comprised 54% of all reservations between August 2020 and May 2021 (Figure 1). After a brief closure due to the COVID-19 pandemic in March 2020, staff revamped the space at the beginning of 2021 to accommodate the Podcasting Suite next to the video-recording equipment already supplied in THE Studio. This allowed the staff to serve a greater volume of students and faculty needing to record audio lectures or podcasts.

**Figure 1:**
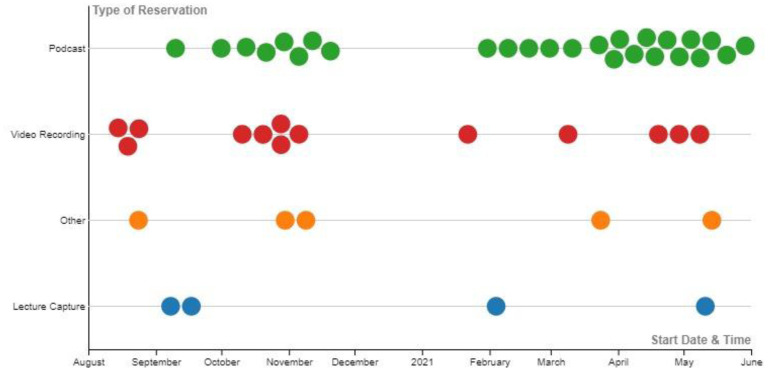
Reservations per month based on reservation type. The Podcasting Suite was introduced in January 2021

Building on previous experiences with creating a low-cost, do-it-yourself multimedia studio in the library, the Podcast Suite was installed within the existing studio set-up [1]. The following equipment was utilized (Figure 2):

- RODECaster Pro
  - A recording device made specifically for amateur podcasting, with features like call-ins, sound effects, and flexible recording.
- Four Electro-Voice RE20 microphones
  - Professional-grade dynamic microphones.
- Four Auray BAI-2X Two-Section Broadcast Arms with integrated fifteen-foot XLR cables
  - Integrated cables that run cleanly to the RODECaster through the desk.
- Four Sony MDR-7506 headphones
  - Popular choice among podcasters, audio studios, and broadcasters.
- Dedicated workstation with desk and chairs

**Figure 2:**
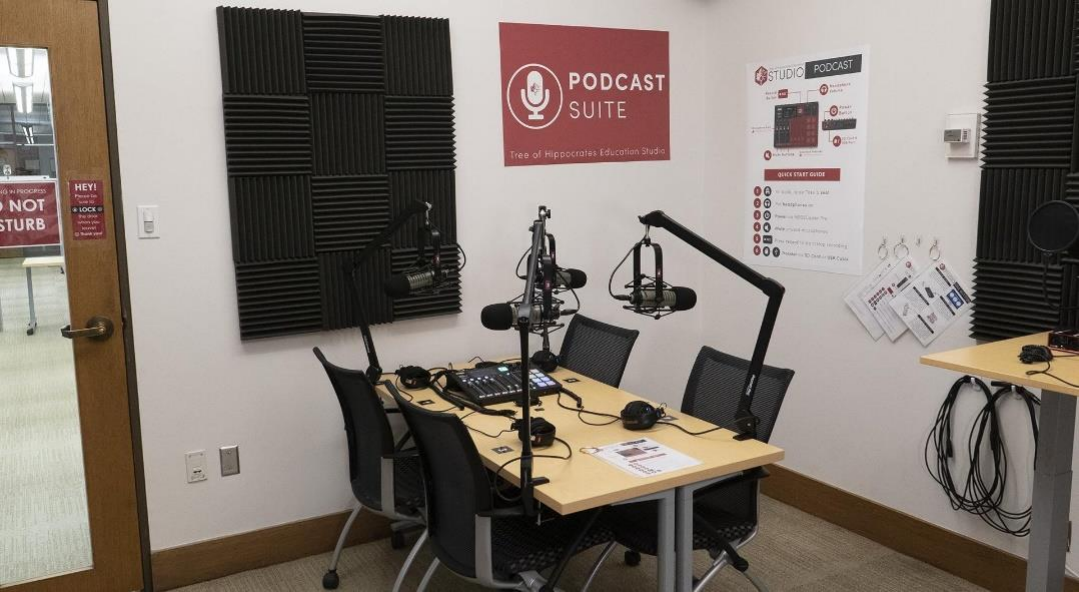
The Podcast Suite inside THE Studio, a multimedia recording space

To familiarize visitors with the equipment in the Podcast Suite, staff created an instructional guide for users. The guide includes:

- Details about the equipment and reserving the space.
- A one-page quick start guide on how to get started on a project.
- Detailed instructions for the RODECaster Pro.
- A tutorial on how to write a podcast script.
- An instructional video on podcast sharing and distribution.

When the Podcast Suite launched in January 2021, staff implemented a simplified reservation system through the Calendly scheduling platform. Users schedule a reservation by choosing a session date, duration, and indicate any special requests or technical assistance they may require. Following every session, Calendly automatically sends a link to a survey to gather feedback about a user's experience.

Podcasts created with the Podcasting Suite are produced and recorded by students, staff, and faculty from the University of Utah's five health sciences schools and colleges, as well as the nearby university hospital. Library staff are available to consult studio users about best practices and ways to incorporate recordings into curriculum. One faculty member, for example, created the *Teaching in Medicine* podcast to supplement an asynchronous trainee-as-teacher program for the Rural Underserved Utah Training Experience (RUUTE) and for resident-as-teacher programs throughout the university. Another faculty member partnered with a fellow dermatologist to cohost a podcast called *Dermasphere*, where the hosts discuss and review recent research that is likely to impact clinical dermatology practice.

Although it's only been a few months since launching the space, library staff have seen regular use from the Podcasting Suite. Staff receive valuable feedback from a survey that is automatically sent to users after their session. A committee of library staff and faculty incorporate survey feedback and usage data into their annual funding requests for upgrades, new equipment, and new programs.

Feedback regarding the Podcast Suite has been positive overall. However, it was noted that some users expressed concerns about the ability to receive technical assistance as there were minimal staff present at the library due to COVID-19. The lack of support issue can be resolved once staff and faculty return to the library on a more permanent basis.

For more information about this project, please reach out to the Eccles Health Sciences Library's shared email address: ehsl-studio@lists.utah.edu.
